# Application of 80 kVp combined with deep learning reconstruction algorithm in overweight patients coronary CT angiography: reduced radiation dose and contrast agent dose

**DOI:** 10.3389/fradi.2026.1876684

**Published:** 2026-08-10

**Authors:** Hangge Pan, Qiushuang Zhang, Jianrong Ding, Jingli Pan, Aiyun Sun

**Affiliations:** 1 Department of Radiology, Taizhou Hospital of Zhejiang Province Affiliated to Wenzhou Medical University, Linhai, China; 2 Key Laboratory of Evidence-Based Radiology of Taizhou, Linhai, China; 3 CT Imaging Research Center, GE HealthCare China, Shanghai, China

**Keywords:** coronary CT angiography, deep learning image reconstruction (DLIR), low tube voltage, overweight, radiation dose

## Abstract

**Background:**

Coronary CT angiography (CCTA) has become a key imaging technique for clinical screening of coronary artery disease (CAD). Overweight patients have a higher prevalence of CAD, however, they face increased risks of radiation exposure and contrast-induced nephropathy during CCTA examinations. This study aimed to explore the feasibility of an 80 kVp scanning protocol combined with deep learning image reconstruction (DLIR) algorithm to reducing radiation dose and contrast agent dose in overweight patients with CCTA.

**Methods:**

A prospective study was conducted on 100 overweight patients who underwent CCTA, randomly divided into two groups of 50 patients each. The scanning tube voltage of group A was 80 kVp, the dose of contrast agent was calculated as BMI×0.14 mL/s×9s, and H-strength DLIR(DLIR-H) was used for image reconstruction. The voltage of group B was 100 kVp, the contrast agent dose was calculated as BMI×0.18 mL/s×9s and the image was reconstruction by 50% adaptive statistical iterative reconstruction-Veo (ASIR-V50%). Radiation dose, contrast agent dose, injection rate, subjective and objective image quality were compared.

**Results:**

Compared to Group B, Group A exhibited significantly lower values for radiation dose (2.85 ± 0.46 vs.4.46 ± 0.69 mSv), contrast agent dose (34.62 ± 2.05 vs. 43.64 ± 1.91 mL) and injection rate (3.83 ± 0.22 vs. 4.84 ± 0.21 mL/s) (all *P* < 0.001), representing reductions of 36.02%, 20.67% and 20.87%, respectively. Inter-reader agreement for subjective scores were excellent (*к* between 0.89 and 1.00). Group A demonstrated significantly lower background noise compared to Group B (15.46 ± 2.82 vs. 21.60 ± 4.36 HU, *P* < 0.001). The signal-to-noise ratio (SNR) and contrast-to-noise ratio (CNR) of major coronary arteries were significantly better in Group A compared to Group B (both *P* < 0.05), except for the SNR of left circumflex artery.

**Conclusions:**

In overweight patients undergoing CCTA, the 80 kVp protocol combined DLIR-H effectively reduced the radiation dose, contrast agent dose and injection rate while ensuring image quality compared to the 100 kVp combined ASIR-V50% scanning protocol.

## Introduction

According to the World Heart Report 2023, over 500 million people worldwide are affected by cardiovascular disease, with 20.5 million deaths attributed to cardiovascular disease in 2021—accounting for nearly one-third of all global deaths (1). Overweight individuals (BMI > 25 kg/m^2^) have a higher incidence of CAD, heart failure, and sudden cardiac death compared to those with normal BMI (2, 3). A report from the World Obesity Federation predicts that by 2035, over 4 billion people will be overweight (4). Consequently, the evaluation of cardiovascular disease in overweight populations is receiving increasing attention. Coronary CT angiography (CCTA) provides both morphological (such as stenosis location and severity) and functional (including fractional flow reserve and plaque characteristics) information about coronary arteries, making it a key imaging modality for CAD screening in clinical practice (5–7).

As the utilization of CCTA continues to rise, so does the cumulative radiation dose, which is particularly concerning for overweight patients. To maintain image quality and diagnostic accuracy, overweight individuals often undergo CCTA with higher tube voltages (8). However, this adjustment leads to an increased radiation dose, consequently elevating the risk of radiation-induced cancer in these patients (9, 10). Furthermore, iodine contrast exhibits lower CT attenuation at high tube voltages, necessitating a higher contrast agent dose to improve tissue contrast, and increasing the risk of contrast-induced nephropathy (11).

The primary method to reducing radiation and contrast agent dose in CCTA is the use of low tube voltage; however, its widespread application is limited due to increased image noise. With continuous advancements in CT technology, the maximum mA capacity of x-ray tubes has been increased, compensating for the reduced x-ray photon energy associated with low tube voltages. This development mitigates image noise and facilitates the implementation of low tube voltage scanning for overweight patients. Moreover, commercially available Deep Learning Image Reconstruction (DLIR) algorithm, integrated into CT scanners has demonstrated superior noise reduction capabilities (12, 13). DLIR provides three selectable levels of reconstruction intensity (DLIR-H, DLIR-M, DLIR-L) to adjust noise reduction. Studies have shown that DLIR-H offers the most effective noise reduction in CCTA; however, these studies were conducted in patients with normal BMI (14, 15). Therefore, this study aims to investigates the feasibility of using 80 kVp combined with DLIR to reduce radiation dose, contrast agent dosage, and injection rate in CCTA examinations of overweight patients.

## Material and methods

### Patient enrollment

**Figure 1:**
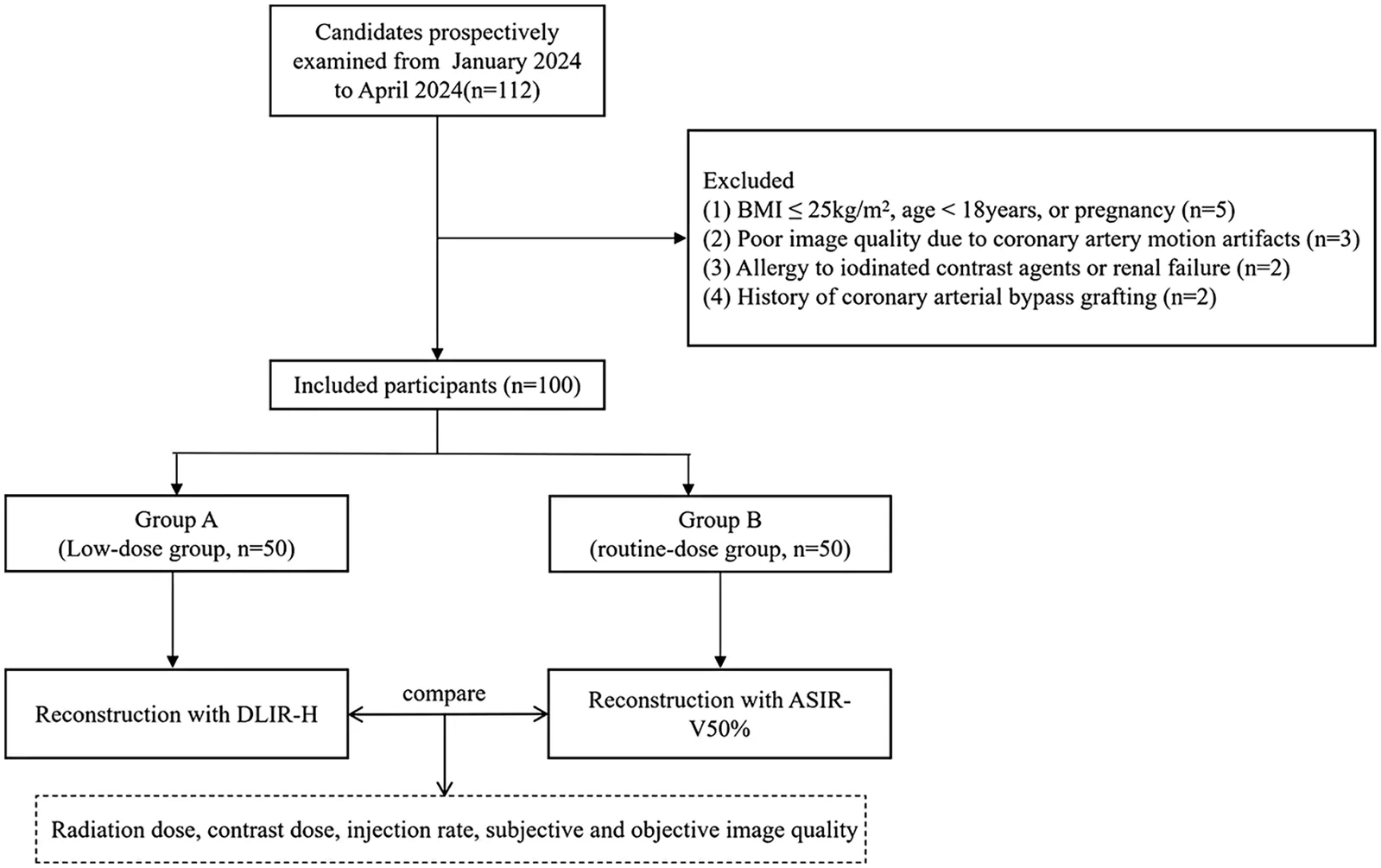
Flowchart of the study design. DLIR-H, deep learning image reconstruction with the high setting; ASIR-V50%, adaptive statistical iterative reconstruction-V with 50% weighting.

This prospective study received official endorsement from the Ethical Committee of Taizhou Hospital of Zhejiang Province (Ethical Approval No. K202603115), and conducted in accordance with the Declaration of Helsinki (as revised in 2013). From January 2024 to April 2024, we prospectively recruited 100 adult patients who underwent CCTA at our hospital and randomly assigned them into groups A and B (see Figure 1). Patients who met any of the following criteria were excluded: (1) BMI ≤ 25 kg/m^2^, age < 18years, or pregnancy; (2) Poor image quality due to coronary artery motion artifacts; (3) Allergy to iodinated contrast agents or renal failure; (4) History of coronary arterial bypass grafting. Informed consent was obtained from all participants.

### Imaging examination

All scans were conducted utilizing a 16 cm wide detector CT scanner (Revolution Apex CT, GE Healthcare) with a prospective axial electrocardiogram (ECG)-triggering protocol. The scan parameters were listed in Table 1. The study protocol did not include supplemental *β*-blocker administration for heart rate (HR) control. A nonionic contrast agent (Iohexol, 350 mgI/mL, GE Healthcare) was administered via a 18–22 G intravenous needle using a high-pressure syringe (CT Motion, Ulrich Medical) into the right median cubital vein, followed by 50 mL saline solution. In Group A (80 kVp), the contrast injection rate was calculated as (mL/s) = BMI×0.14, with an injection duration of 9 s and a trigger threshold of 220 HU. In Group B (100 kVp), the injection rate was (mL/s) = BMI×0.18, with the same injection duration and trigger threshold. Prior to the enhanced CCTA, all patients underwent an unenhanced ECG-gated coronary calcium scan using identical scanning parameters, including a prospectively triggered acquisition at 75% of the R-R interval, for coronary artery calcification scoring.

**Table 1:** Scanning parameters.

Parameter	Group A	Group B
Tube voltage (kVp)	80	100
Tube current (mA)	500–1,300*	500–1,300*
Noise index	15	15
Rotation time (sec)	0.28	0.28
Detector collimation (mm)	256*0.625	256*0.625
Slice thickness/interval (mm)	0.625/0.625	0.625/0.625

Group A, Low-dose group; Group B, routine-dose group.

*Automatic tube current modulation (ATCM).

### Image post-reconstruction

All images were automatically processed to determine the optimal temporal phase of the cardiac cycle using intelligent cardiac phase image selection technology (Smart Phase technology, GE Healthcare), and coronary artery motion was corrected using a motion correction algorithm (Snapshot Freeze 2, SSF2, GE Healthcare). In Group A, image reconstruction was performed using DLIR-H, while in Group B, images were reconstructed using adaptive statistical iterative reconstruction-Veo at a level of 50% (ASIR-V50%). All unenhanced images were transferred to commercial artificial intelligence software (CoronaryDoc Plus, ShuKun Technology, Beijing, China) for coronary artery calcification scoring. Additionally, all enhanced images were processed using the same software to generate maximum intensity projection, multiplanar reformation, curved planar reformation, and volume rendering images.

### Image quality evaluation

The CT attenuation and standard deviations (SD) of each coronary artery segment were measured. ROIs were placed at the aortic root (AR), proximal right coronary artery (RCA), left anterior descending artery (LAD) and left circumflex artery (LCX), with respective ROI areas of 50 mm², 2 mm², 2 mm², and 2 mm². The SD of chest wall fat (FAT) at the AR level was measured as background noise for cardiac images, with an ROI areas of 10 mm². All ROIs were positioned at the center of the target artery while avoiding the arterial wall, plaque, and severe artifacts. We randomly resampled 20 out of 100 patients for repeated measurements to test the reliability of our quantitative data. SNR and CNR were calculated as follows: SNR = CT_artery_/SD_artery_, CNR = (CT_artery_−CT_fat_)/SD_fat_.

Image sharpness was evaluated using the edge rise slope (ERS) and edge rise distance (ERD) at AR in the axial plane (16, 17). The starting point was set at the center of the artery, and equidistant extension lines were drawn outward to generate CT attenuation curves using Image J software (National Institutes of Health, Bethesda, MD) [http://rsb.info.nih.gov/ij] and its particle analysis tool (Plot Profile). The 10%–90% range of the ERD from the attenuation curve was selected to define the width of the edge response at the boundary. The ERS was calculated as follows: ERS = (CT90% - CT10%)/ERD, (see Supplementary Figure S1).

Two radiologists (one senior and one junior) independently assessed image quality using a five-point Likert scale, with scores of 3–5 being considered acceptable. The radiologists were blinded to the study groups and evaluated images based on arterial edge sharpness, clarity of small arterial details, degree of venous contamination, artifacts and image noise. The scoring criteria were as follows: 5 for excellent, 4 for good, 3 for acceptable, 2 for poor, and 1 for very poor image quality. In case of disagreement, the final score was reached by consensus.

The degree of coronary artery stenosis was classified as no stenosis (0%), minimal stenosis (1%–24%), mild stenosis (25%–49%), moderate stenosis (50%–69%), severe stenosis (70%–99%), and occluded (100%) by two radiologists (one senior and one junior) according to the Coronary Artery Disease Reporting and Data System (CAD-RADS™) (18). In case of disagreement, the final classification was determined by the senior radiologist. The calcification score for each coronary artery was recorded from the AI software, and the severity of coronary artery calcification was categorized based on Agatston score (19): no calcified (0), minimal calcified (0–10), mild calcified (11–100), moderate calcified (101–400) and severe calcified (>400).

### Radiation dose

The CTDIvol (volume CT dose index, mGy) and DLP (dose length product, mGy·cm) values, which are automatically recorded by the scanner after each scan, were documented. The CTDIvol represents the average radiation dose per slice across the entire scan volume, while the DLP measures the radiation burden during a single examination. DLP was calculated as follows: DLP = CTDIvol×L (with L representing the scan length along the Z-axis). The effective radiation dose (ED, mSv) was performed by multiplying the DLP with a specific factor k (for coronary imaging, k = 0.014 mSv·mGy^−1^·cm^−1^) (20). Both DLP and ED collectively provided a comprehensive assessment of patient-specific radiation exposure.

### Statistical analysis

In this study, SPSS 26.0 statistical software (IBM Corp.) was used for statistical analysis. The normality of continuous variables was assessed using the Shapiro–Wilk test. Normally distributed continuous variables are presented as mean ± standard deviation (SD) and were compared between the two groups using the independent-samples *t* test. Non-normally distributed continuous variables were compared using the Mann–Whitney *U* test. Subjective image quality scores, which were measured on a five-point Likert scale, were also analyzed using the Mann–Whitney *U* test. Categorical variables were compared using Fisher's exact test. Inter-reader agreement for subjective image quality assessment was evaluated using Cohen's *κ* coefficient (a *κ* value over 0.8 is classified as excellent, between 0.61 and 0.8 as good, and less than 0.6 as poor). All statistical tests were two-sided, and a *P* value < 0.05 was considered statistically significant. Sample size estimation was performed using G*Power.

## Results

### Participants

As shown in Table 2, all 100 enrolled patients (66 males and 34 females) successfully completed the CCTA examination. There were no significant differences in age between the two groups (56.22 ± 10.17 vs. 57.52 ± 10.50 years, *P* = 0.53), BMI (27.27 ± 1.54 vs. 27.02 ± 1.28 kg/m^2^, *P* = 0.38), HR [70.12 ± 11.84 vs. 71.92 ± 13.74 beat per minute (bpm), *P* = 0.48] or other baseline characteristics. However, the difference of radiation dose, contrast agent dose and injection rate between the two groups was statistically significant (all *P* < 0.001). Compared with Group B, Group A demonstrated: a 36.02% reduction in radiation dose (2.85 ± 0.46 vs. 4.46 ± 0.69 mSv), a 20.67% reduction in contrast agent dose (34.62 ± 2.05 vs. 43.64 ± 1.91 mL), and a 20.87% reduction in injection rate (3.83 ± 0.22 vs. 4.84 ± 0.21 mL/s).

**Table 2:** Patient characteristics.

Patient characteristics	Group A [95%CI]	Group B [95%CI]	*P* value
Age (years)	56.22 ± 10.17	57.52 ± 10.50	0.53
Gender (male: female)	31: 19	35: 15	0.40
Weight (kg)	74.45 ± 8.16	74.86 ± 8.26	0.80
Height (m)	1.65 ± 0.07	1.66 ± 0.08	0.43
BMI (kg/m^2^)	27.27 ± 1.54	27.02 ± 1.28	0.38
HR (bpm)	70.12 ± 11.84	71.92 ± 13.74	0.48
Contrast dosage (mL)	34.62 ± 2.05 [34.04–35.20]	43.64 ± 1.91 [43.10–44.18]	<0.001
Injection rate (mL/s)	3.83 ± 0.22 [3.77–3.89]	4.84 ± 0.21 [4.78–4.90]	<0.001
DLP (mGy*cm)	203.86 ± 33.10 [194.46–213.27]	318.62 ± 49.34 [304.60–332.64]	<0.001
ED (mSv)	2.85 ± 0.46 [2.72–2.99]	4.46 ± 0.69 [4.26–4.66]	<0.001

Data are presented as the mean ± standard deviation; 95%CI, 95% Confidence Interval.

Group A, Low-dose group; Group B, routine-dose group.

BMI, body mass index; HR, heart rate; DLP, dose–length product; ED, effective dose.

Coronary artery stenosis was identified in 54 patients, and 49 patients demonstrated coronary artery calcification. Comparative analysis showed no statistically differences in stenosis (*P* = 0.39) or calcification (*P* = 0.61) between two groups.

### Objective evaluation

**Figure 2:**
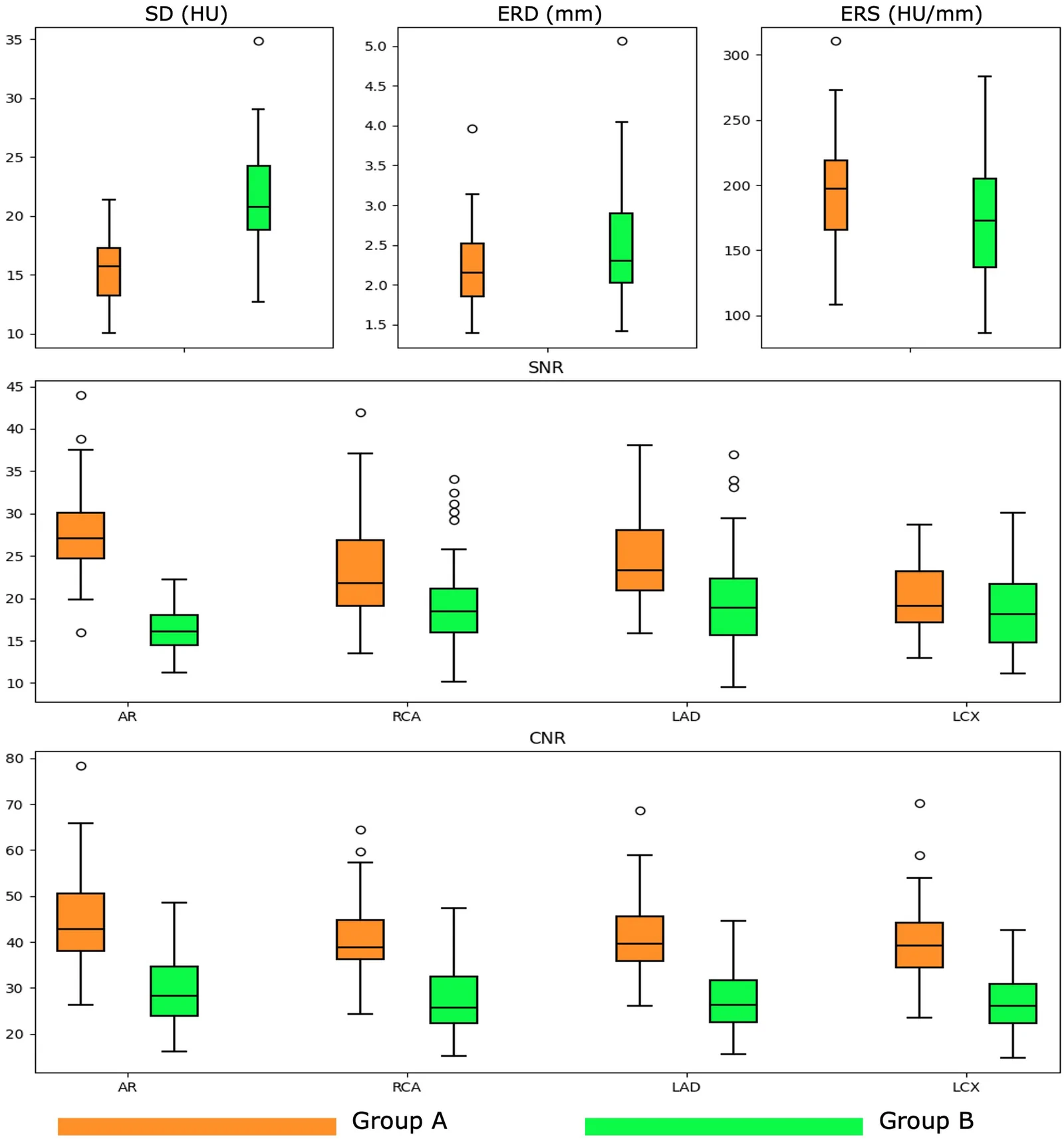
Comparison of the objective evaluation of each artery. AR, aortic root; RCA, right coronary artery; LAD, left anterior descending artery; LCX, left circumflex artery; SD, standard deviation; ERD, edge rise distance; ERS, edge-rise-slope; SNR, signal-to-noise ratio; CNR, contrast-to-noise ratio; Group A, Low-dose group; Group B, routine-dose group.

The CT attenuations of all segmented arteries were significantly higher in Group A than in group B (all *P* < 0.05). Additionally, background noise in Group A was lower than in Group B (15.46 ± 2.82 HU vs. 21.60 ± 4.36 HU, *P* < 0.001). The consistency of objective data measurements is shown in Supplementary Table S1. Except for the LCX (SNR_A−LCX_ vs. SNR_B−LCX_, *P* = 0.09), the SNR of all other segmented arteries was significantly higher in Group A than in group B (SNR_A−AR_ vs. SNR_B−AR_, *P* < 0.001; SNR_A−RCA_ vs. SNR_B−RCA_, *P* = 0.004; SNR_A−LAD_ vs. SNR_B−LAD_, *P* < 0.001). The CNR of all segmented arteries was also significantly higher in Group A than in Group B (all *P* < 0.001). Regarding image sharpness, the ERS in Group A was significantly higher than that in Group B, while ERD was significantly lower than that in Group B (ERS_A_ vs. ERS_B_, *P* = 0.02; ERD_A_ vs. ERD_B_, *P* = 0.02). See Table 3 and Figure 2 for detailed results.

**Table 3:** Objective image quality comparison.

Locations	Group A	Group B	*P* value
CT(HU)			
AR	553.37 ± 77.99	510.78 ± 64.08	0.004
RCA	495.86 ± 65.90	457.96 ± 58.43	0.003
LAD	501.19 ± 64.36	463.56 ± 66.18	0.005
LCX	483.27 ± 68.59	449.81 ± 58.32	0.01
SD (HU)			
FAT	15.46 ± 2.82	21.60 ± 4.36	<0.001
SNR			
AR	27.43 ± 5.11	16.20 ± 2.39	<0.001
RCA	22.93 ± 6.33	19.50 ± 5.23	0.004
LAD	24.18 ± 5.12	19.81 ± 5.94	<0.001
LCX	20.30 ± 4.34	18.74 ± 4.83	0.09
CNR			
AR	44.64 ± 9.51	29.70 ± 7.29	<0.001
RCA	40.85 ± 8.50	27.18 ± 6.88	<0.001
LAD	41.19 ± 8.47	27.37 ± 6.74	<0.001
LCX	40.03 ± 8.65	26.69 ± 6.32	<0.001
ERS	194.95 ± 42.28	173.65 ± 47.33	0.02
ERD	2.21 ± 0.53	2.51 ± 0.74	0.02

Data are presented as the mean ± standard deviation. AR, aortic root; RCA, right coronary artery; LAD, left anterior descending artery; LCX, left circumflex artery; SD, standard deviation; SNR, signal-to-noise ratio; CNR, contrast-to-noise ratio; Group A, Low-dose group; Group B, routine-dose group; ERS, edge-rise-slope; ERD, edge rise distance.

### Subjective evaluation

**Figure 3:**
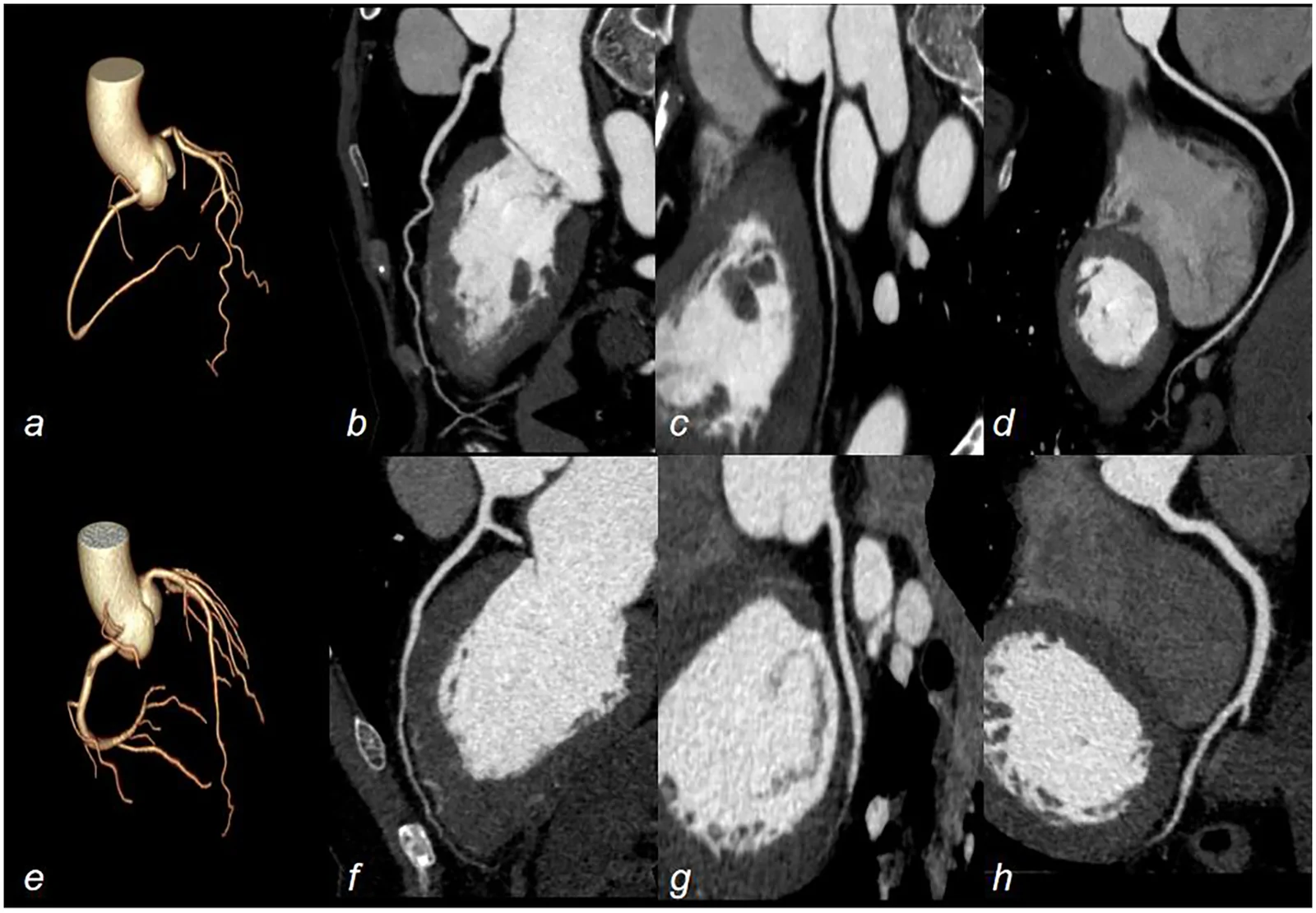
Image quality comparison between the two groups. **(a–d)** are reconstructed images from Group A. The patient is a 53-year-old female with a BMI of 26.35 kg/m² and a HR of 70 bpm. **(e)**-**(h)** are reconstructed images from Group B. The patient is a 38-year-old male with a BMI of 26.45 kg/m² and a HR of 62 bpm. There was no statistically significant difference on subjective quality score between the two groups. The images in group A had sharper anatomical details, smoother artery edges, and fewer artifacts than group B.

Both radiologists assigned subjective image quality scores ≥ 3 for all images in both groups, indicating that the images met diagnostic requirements. The subjective quality scores were significantly higher in Group A than those in Group B for all evaluated arteries (*P*_RCA_ = 0.001, *P*_LAD_ < 0.001, *P*_LCX_ < 0.001). This scanning protocol is unsuitable for examining patients with myocardial bridges, so we excluded those with a history of coronary arterial bypass grafting (See Table 4 and Figure 3).

**Table 4:** Subjective image quality scores.

SIQS	Group A (*n* = 50)		Group B (*n* = 50)		*P* value
	Reader 1	Reader 2	Reader 1	Reader2	
RCA					0.001
5	29	27	18	16	
4	20	22	28	31	
3	1	1	4	3	
2	0	0	0	0	
1	0	0	0	0	
Kappa Score	0.92		0.89		
LAD					<0.001
5	17	15	6	7	
4	29	31	32	32	
3	4	4	12	11	
2	0	0	0	0	
1	0	0	0	0	
Kappa Score	0.92		0.92		
LCX					<0.001
5	17	19	6	7	
4	30	29	36	34	
3	3	2	8	9	
2	0	0	0	0	
1	0	0	0	0	
Kappa Score	0.88		0.91		

SIQS, subjective image quality score; Group A had significantly higher subjective image quality scores than Group B (*P* < 0.001); RCA, right coronary artery; LAD, left anterior descending artery; LCX, left circumflex artery.

## Discussion

Our study demonstrated that in patients with BMI > 25 kg/m², the use of 80 kVp combined with DLIR-H in CCTA resulted in a 36.02% reduce radiation dose, a 20.67% reduction in contrast agent dose and a 20.87% reduction in injection rate compared with the 100-kVp protocol and ASIR-V50%. Additionally, this approach yielded lower image noise, higher SNR, higher CNR, and improved subjective quality scores.

In recent years, reducing radiation dose in overweight patients while maintaining image quality has been a key area of research. Overweight individuals typically have a high fat content, which increases x-ray photon attenuation and scattering, leading to elevated image noise. To compensate for this, higher tube voltages are often required, which consequently results in higher radiation exposure per examination. Moreover, individuals with a high BMI face an increased risk of CAD (21), necessitating more frequent repeated examinations and thus accumulating a higher radiation dose.

Conventional iterative reconstruction algorithms can reduce radiation dose while preserving image quality to some extent. However, in the process of noise reduction or contrast enhancement, these algorithms often overprocess image details, potentially leading lose its natural texture and structural layering. In this study, an algorithm (DLIR) based on deep convolutional neural network modeling is applied. This algorithm utilizes low-dose projected images as input data and high-dose filtered back projection (FBP) reconstructed images as target data, training a computational model to optimize reconstruction parameters. Compared to conventional iterative algorithms, DLIR incorporates millions of adjustable parameters, significantly enhancing its performance. It effectively reduces image noise while preserving or even improving anatomical details visualization, resulting in sharper and more realistic images (13). This advancement enables low-radiation CCTA scans in overweight patients using low tube voltage, thereby expanding the applicability of low-dose imaging to this population. Additionally, according to x-rays physics, the attenuation coefficient of iodine increases as tube voltage decreases. Consequently, lower contrast agent doses can be used in low-kVp scans while maintaining the same CNR, thereby reducing the risk of contrast-induced nephropathy. The findings of this study support the aforementioned advantages: the SNR and CNR of all coronary artery segments in Group A were superior to those in Group B. Although the intergroup difference in LCX SNR failed to reach statistical significance (*P* = 0.09), the *P* value was close to 0.05. This phenomenon is presumed to be associated with the limited sample size or the small luminal diameter of the LCX. Larger sample cohorts should be enrolled in future studies to further validate this observation.

Previous studies have demonstrated the feasibility of low tube voltage scanning with reduced contrast dosage in overweight patients. Li et al. (22) compared 80 kVp with 120 kVp, demonstrating a 45.2% reduction in radiation dose (1.01 ± 0.45 vs. 1.85 ± 0.40 mSv) and a 43% reduction in contrast agent dose (33.69 ± 3.87 vs. 59.11 ± 5.60 mL). Similarly, Wang et al. (23) compared 100 kVp with 120 kVp, demonstrating a 40% reduction in radiation dose (1.6 ± 0.1 vs. 1.0 ± 0.1 mSv). In our study, compared 80 kVp with 100 kVp, leading to a 36% reduction in radiation dose (2.85 ± 0.46 vs. 4.46 ± 0.69 mSv) and a 21% reduction in contrast agent dose (34.62 ± 2.05 vs. 43.64 ± 1.91 mL). The variation in radiation dose reduction across studies may be attributed to differences in scanning protocols. To compensate for the reduced x-ray photon energy at lower tube voltages, we employed a smaller noise index (NI-coronary = 15) and a wider mA scanning range (100–1,300 mA). Additionally, to provide insights into the dynamic changes in cardiac structure and function across different phases of the cardiac cycle, thereby enhancing diagnosis and treatment of cardiovascular diseases, we utilized a full cardiac cycle scanning mode (24). However, this approach inherently results in higher radiation exposure.

In the studies by Santis et al. (25) and Zhu et al. (26), patients with HR greater than 90 bpm were excluded. Similarly, Dominik et al. (27) required medication to reduce HR above 65 bpm before imaging. In contrast, our study utilized the second-generation motion correction algorithm (Snapshot Freeze 2, SSF2), to correct coronary motion. This algorithm effectively maintains high image quality even in patients elevated heart rates or arrhythmias, without being limited by HR or respiration. In our study, patient HRs ranged from 44 to 115 bpm, with 11 patients exhibiting HR greater than 90 bpm or arrhythmias. Given its robustness in motion correction, our study protocol can be applied to a broader patient population and is well-suited for clinical implementation.

Several limitations of this study should be acknowledged: (1) This study focus on comparing image quality, radiation dose, contrast agent dosage and injection rate in CCTA, but did not evaluating accuracy against digital subtraction angiography, the gold standard. (2) Our BMI range was 25 kg/m² to 31.77 kg/m², the applicability of these findings to patients with higher BMI requires further investigation. (3) This scanning protocol is unsuitable for examining patients with myocardial bridges, so we excluded those with a history of coronary arterial bypass grafting. (4) The primary objective of this study was to compare the comprehensive clinical performance of two complete scanning protocols, rather than performing single-factor comparative analysis for individual scanning parameters.

## Conclusion

In CCTA examinations of overweight patients, the 80-kVp combined DLIR-H protocol significantly reduced radiation dose, contrast agent dose and injection rate while maintaining image quality, compared to the 100-kVp combined ASIR-V50% protocol.

## Data Availability

The raw data supporting the conclusions of this article will be made available by the authors, without undue reservation.

## Supplementary material

Supplementary Table S1 Measurement consistency of objective data.

Supplementary Figure S1 Examples of quantitative measurement used in the study. (a) Draw a perpendicular line on the target vessel to generate a CT attenuation profile in panel b. (b) The blue dots are the last point of the profile before a rapid descent and the first point that tends to level off, and two red dots are 10% and 90% of the height difference between the blue points on the CT attenuation profile, respectively. The ERS is defined as the slope between two red dots, the ERD is defined as the change in the horizontal coordinate between two red dots. ERS, edge-rise-slope; ERD, edge rise distance.
