# Supplementary material for: Application of 80 kVp combined with deep learning reconstruction algorithm in overweight patients coronary CT angiography: reduced radiation dose and contrast agent dose
Source: Front Radiol. 2026 Aug 10;6:1876684. doi: 10.3389/fradi.2026.1876684 (PMC13501517; doi:10.3389/fradi.2026.1876684)
Supplement: Supplementary file 1 [file Table1.doc]

Table S1 Measurement consistency of objective data

| Locations | Measurement consistency [*95%CI*] |
| --- | --- |
| CT(HU) |  |
| FAT | 0.98 [0.94 to 0.99] |
| AR | 0.96 [0.91 to 0.98] |
| RCA | 0.96 [0.91 to 0.98] |
| LAD | 0.95 [0.87 to 0.98] |
| LCX | 0.93 [0.84 to 0.97] |
| SD (HU) |  |
| FAT | 0.94 [0.85 to 0.98] |
| AR | 0.99 [0.96 to 0.99] |
| RCA | 0.89 [0.74 to 0.95] |
| LAD | 0.94 [0.87 to 0.98] |
| LCX | 0.96 [0.91 to 0.98] |

95%CI, 95% Confidence Interval; AR, aortic root; RCA, right coronary artery; LAD, left anterior descending artery; LCX, left circumflex artery; SD, standard deviation.
